# A new trial design to accelerate tuberculosis drug development: the Phase IIC Selection Trial with Extended Post-treatment follow-up (STEP)

**DOI:** 10.1186/s12916-016-0597-3

**Published:** 2016-03-23

**Authors:** Patrick P. J. Phillips, Kelly E. Dooley, Stephen H. Gillespie, Norbert Heinrich, Jason E. Stout, Payam Nahid, Andreas H. Diacon, Rob E. Aarnoutse, Gibson S. Kibiki, Martin J. Boeree, Michael Hoelscher

**Affiliations:** MRC Clinical Trials Unit at University College London, Aviation House, 125 Kingsway, London, WC2B 6NH UK; School of Medicine, Johns Hopkins University, Baltimore, Maryland USA; School of Medicine, University of St. Andrews, St. Andrews, UK; Division of Infectious Diseases and Tropical Medicine, Medical Center of the Ludwig-Maximilians-University (LMU), Munich, Germany; German Center for Infection Research (DZIF), Munich Partner Site, Munich, Germany; School of Medicine, Duke University, Durham, North Carolina USA; University of California San Francisco, San Francisco, California USA; Division of Physiology, Department of Medical Biochemistry, Stellenbosch University, Tygerberg, South Africa; TASK Applied Science, Bellville, South Africa; Department of Pharmacy, Radboud University Medical Center, Nijmegen, The Netherlands; East African Health Research Commission (EAHRC), East African Community, Arusha, Tanzania; Department of Lung Diseases, Radboud University Medical Center, UCCZ Dekkerswald, Nijmegen, The Netherlands

**Keywords:** Tuberculosis, Clinical trials, Middle development, Phase IIC, STEP, Drug development, Regimen development

## Abstract

**Background:**

The standard 6-month four-drug regimen for the treatment of drug-sensitive tuberculosis has remained unchanged for decades and is inadequate to control the epidemic. Shorter, simpler regimens are urgently needed to defeat what is now the world’s greatest infectious disease killer.

**Methods:**

We describe the Phase IIC Selection Trial with Extended Post-treatment follow-up (STEP) as a novel hybrid phase II/III trial design to accelerate regimen development. In the Phase IIC STEP trial, the experimental regimen is given for the duration for which it will be studied in phase III (presently 3 or 4 months) and patients are followed for clinical outcomes of treatment failure and relapse for a total of 12 months from randomisation. Operating characteristics of the trial design are explored assuming a classical frequentist framework as well as a Bayesian framework with flat and sceptical priors. A simulation study is conducted using data from the RIFAQUIN phase III trial to illustrate how such a design could be used in practice.

**Results:**

With 80 patients per arm, and two (2.5 %) unfavourable outcomes in the STEP trial, there is a probability of 0.99 that the proportion of unfavourable outcomes in a potential phase III trial would be less than 12 % and a probability of 0.91 that the proportion of unfavourable outcomes would be less than 8 %. With six (7.5 %) unfavourable outcomes, there is a probability of 0.82 that the proportion of unfavourable outcomes in a potential phase III trial would be less than 12 % and a probability of 0.41 that it would be less than 8 %. Simulations using data from the RIFAQUIN trial show that a STEP trial with 80 patients per arm would have correctly shown that the Inferior Regimen should not proceed to phase III and would have had a high chance (0.88) of either showing that the Successful Regimen could proceed to phase III or that it might require further optimisation.

**Conclusions:**

Collection of definitive clinical outcome data in a relatively small number of participants over only 12 months provides valuable information about the likelihood of success in a future phase III trial. We strongly believe that the STEP trial design described herein is an important tool that would allow for more informed decision-making and accelerate regimen development.

## Background

New regimens are urgently needed for the treatment of tuberculosis (TB). The current standard of care, a 6-month four-drug regimen, has been in use for decades. Under optimal clinical trial conditions it can achieve a durable cure in 92 % of patients [1]; in practice, approximately 10 % of patients are forced to stop treatment prematurely owing to toxicity or because life circumstances present challenges to prolonged adherence to supervised treatment. Many of these patients will later experience relapse and, under programme settings, completion rates (a proxy for cure) are closer to 80–85 % [2]. Thus, the standard regimen is inadequate to control the epidemic and shorter, simpler regimens are needed to defeat what is now the world’s greatest infectious disease killer [2].

Exciting progress has been made, with two compounds in different classes (bedaquiline and delamanid) receiving accelerated approval by regulators for the treatment of multi-drug resistant TB (MDR-TB) since 2012 [2]. In addition, there has been growing interest in optimising old TB drugs [3] and repurposing drugs with other indications that were previously thought to have limited activity against TB [4, 5]. A number of clinical trials of shorter regimens combining new and old drugs are ongoing (ClinicalTrials.gov identifiers NCT02410772, NCT02581527 and NCT02342886). Innovative trial designs originally developed in oncology, including the multi-arm multi-stage design (MAMS) that allows for poorly performing arms to be stopped early, have been implemented in TB phase II trials with some success [6, 7]. There has, however, been no change in the treatment of drug-sensitive TB for more than 40 years. Most recently, three major international multicentre phase III trials demonstrated that four different 4-month regimens did not provide as good a standard of care as the 6-month regimen [1, 8, 9].

Antibiotics, and in particular those used for the treatment of TB, were some of the first drugs evaluated systematically in medicine. While establishing the composition of anti-TB first-line treatment is often thought to be the first example of evidence-based medicine or ‘rational therapeutics’ [10, 11], this feat was achieved largely empirically through a series of studies that we would now define as phase III trials [12]. Thus, there has been no established pathway for clinical development in TB, and the approach put forward by drug developers has been instituted only in the last 10–15 years. In fact, no new compound has been approved for the treatment of drug-sensitive TB since the disease was declared a global emergency by the World Health Organization in 1993, with the exception of intermittent low-dose rifapentine in the continuation phase of treatment [13], which has been implemented only in a few low-incidence settings. The accelerated approvals of the two new drugs for MDR-TB, bedaquiline and delamanid, were based on data from phase II trials only. This was possible because these trials employed surrogate endpoints that were considered likely to reflect clinical outcomes [14] and because MDR-TB treatment outcomes with the current standard of care are so poor (54 % cure [15]) and the need for new medicines so acute. Full approval will only be achieved once phase III trials are completed successfully. The accelerated approval framework with the US and EU regulators could not be used for drug-sensitive TB because there is an established and highly effective, albeit prolonged, treatment. Furthermore, such accelerated approval mechanisms are not often available for many regulators outside the US and the EU.

Clinical development of new treatments is conventionally split into three phases. Phase I trials are first-in-human studies aimed at exploring safety, pharmacokinetics, and relevant drug-drug interactions [16]. The safety of the participants (often healthy volunteers) is the overwhelming primary concern because incidence and the magnitude of toxicities cannot be readily predicted by preclinical toxicology studies, and unexpected serious adverse events not seen in animal studies do sometimes occur [17]. Phase III trials are large and expensive multicentre randomised studies that are designed to provide confirmatory evidence that a new treatment is safe and efficacious. Thus, the objectives of the phase III trial are to provide adequate evidence to convince regulators to approve the treatment for the disease in question and to change policy and practice. In between these two poles of development is a critical area: phase II or ‘middle development’ [18], where the treatment is given to individuals with the target disease with the objectives of identifying an optimal therapeutic dose or doses, exploring and establishing safety in a larger group of participants, and generating efficacy data (often on intermediate endpoints) sufficient to provide the confidence needed to justify a phase III trial. Because TB is treated with regimens rather than individual drugs, an additional goal of middle development is to optimise and select those combinations with the highest likelihood of success in phase III treatment-shortening trials.

Middle development for TB drugs typically follows a sequential two-step or three-step pathway. The first step is a 14-day monotherapy trial (a new drug can only be given for a maximum of 14 days as monotherapy owing to the risk of acquiring drug resistance), which is used to demonstrate the microbiological activity of the agent alone at different doses. This is often followed by a 7-day to 14-day study of drugs in combination. These trials are followed by longer-duration studies of drugs in combination (traditionally 8 weeks). An example of a development pathway is that of pretomanid (PA-824), first evaluated as monotherapy, then in combination in 14-day and 8-week studies [19–23]. The 14-day studies are considered phase IIA and 8-week studies as phase IIB, and in each case all patients are given standard treatment after completing the experimental therapy so that the total duration of treatment is not less than the standard 6 months. The primary endpoints of such studies are microbiological intermediate endpoints, that is, measuring the speed at which bacilli are killed using serially collected sputum during the first days and weeks of treatment, and include the decline in the number of colony-forming units growing on solid culture media [23], increase in time to positivity in liquid culture media [23], time to stable negative culture conversion [3], or proportion of participants with negative sputum cultures at a specific time during treatment [24]. Killing bacilli during a course of treatment is a complex process that is not well understood. It has been proposed that different drugs act on up to four different sub-populations of bacilli, because drug activity may depend on the metabolic state of the organism and the compartment in which the organism resides in the infected host [25]. Some drugs, such as isoniazid, are thought to act earlier, targeting metabolically active, rapidly multiplying organisms. Others, such as pyrazinamide and rifampicin, are believed to act later, when the hardest-to-kill bugs must be eradicated. Penetration of different drugs into the lesions where the bacilli reside is another area that affects the timing and extent of drug activity [26]. For this reason, there is limited value in only measuring the activity of a dose or a regimen during the first 14 days to identify the combinations to advance from phase IIA to phase IIB studies [27], and the activity may depend on the drug’s mechanism of action and pharmacokinetic characteristics. For example, only a small effect was seen with bedaquiline in an initial 7-day study [28], but in a follow-on 14-day trial that included loading doses, clear activity was demonstrated [29]. Clofazimine had no effect over 14 days at tested doses [22]. Nevertheless, both bedaquiline and clofazimine have important roles in combination with other agents in longer duration randomised clinical trials for the treatment of MDR-TB [30, 31]. In practice, the 14-day and 8-week studies merely demonstrate that the drug or regimen has some anti-mycobacterial activity in humans, but both yield incomplete efficacy or safety data. More importantly, even microbiological activity over 8 weeks has a limited role in decision-making for advancing regimens to phase III trials [32–35]. The modest benefits in microbiological activity with moxifloxacin seen in 8-week studies were confirmed in the REMoxTB phase III trial [1], but these benefits did not translate into activity sufficient to cure patients in just 4 months. Stated directly, many regimens with impressive microbiological activity over 2 or 8 weeks of treatment are not sufficiently active to allow for treatment shortening, demonstrated by high rates of relapse [1, 8, 9, 12]. Furthermore, safety data generated in phase II trials are valuable but limited given the shorter duration of use over only 8 weeks.

The model of clinical development employed by the pharmaceutical industry assumes a limited number of drug candidates and is focused on gathering adequate data to inform go/no-go decisions along the development pathway so that promising compounds are advanced efficiently and unpromising ones are abandoned early. In general, about 60–70 % of drugs that enter phase III trials are successfully licensed, but the success rate depends highly on the disease entity and drug class [36]. This model has informed many of the common approaches in TB regimen development, with doses and regimens selected based on early clinical studies and carried through the development process until they fail. This pathway for developing regimens for TB has not been successful to date. Because TB is a major public health problem with drug development funded largely through public and philanthropic sources [37], the focus of middle development should be the efficient identification and testing of those regimens that are most likely to shorten TB therapy rather than excessive attention to particular drugs or combinations. A barrier to this is the panoply of potential old, repurposed, and new drugs, which results in a large number of possible three-drug and four-drug combination regimens that might lead to shorter, simpler regimens.

To redress this over-reliance on 14-day and 8-week endpoints and to facilitate this broader perspective on the middle development of TB drugs and regimens, we propose a new hybrid phase II/III trial design: the Phase IIC Selection Trial with Extended Post-treatment follow-up (STEP). We believe that the Phase IIC STEP trial design will help to bridge the gap between phase II and phase III by improving the quality and relevance of data generated in middle development. This improved evidence base prior to phase III will allow for informed decisions for selecting novel regimens to proceed to confirmatory trials and will reduce the risk of expensive phase III failures. In this paper we describe the STEP design and explore its operating characteristics under typical scenarios. We have also conducted a simulation study using data from a large, recently completed phase III trial to illustrate how such a design could be used in practice.

## Methods

### STEP trial design

We define a STEP trial as one with the following features:

1. The novel regimen is given for the duration for which it is intended to be studied in phase III (presently 3 or 4 months).
2. Treatment is stopped after experimental therapy is completed (provided there is sufficient confidence that it is safe to do so from evidence collected in the study itself or in previous studies).
3. Patients are followed for clinical outcomes of treatment failure and relapse for a total of 12 months from randomisation.

**Fig. 1:**
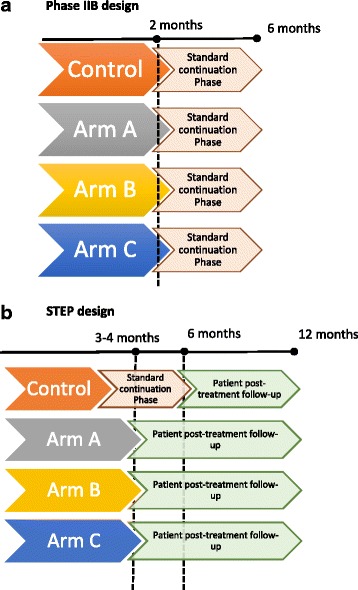
Comparison of standard phase IIB design (**a**) with the STEP design (**b**)

This can be contrasted with the current phase IIB design (Fig. 1). A STEP trial is designed with a similar sample size to a standard phase IIB study with the primary outcome defined in typical fashion—time to culture conversion or decline in bacillary load over time [23, 38]. However, because the regimen is given for its intended duration, and patients are followed for a total of 12 months from randomisation, data on the definitive clinical endpoint, status at 12 months post-randomisation (a composite outcome of treatment failure and relapse) is also collected. This composite outcome is the same as that used in confirmatory phase III trials [1, 8]. Twelve months of follow-up is considered adequate because more than 75 % of relapses occur within 6 months of stopping treatment [39]; 18 months of follow-up would considerably prolong the duration of the trial without significantly enhancing the knowledge gained. While the precision in this composite unfavourable outcome will be low because unfavourable outcomes are rare and phase II trials are relatively small (and it therefore should formally be a secondary rather than primary outcome in the trial), it will provide invaluable information to guide decisions about further clinical development of the regimen. Equally important are the longer-term safety and tolerability data, both during treatment and following completion of treatment. A STEP trial could be considered as a pilot phase III trial, with the collection of similarly rich data on clinically relevant outcomes but at a much lower sample size and cost. Results of a STEP trial may support several potential development strategies: direct advancement for evaluation in a definitive phase III trial, further phase II work to optimise the doses or change the regimen components, pharmacokinetic-pharmacodynamic (PK-PD) assessments to understand underperforming regimens or identify populations that are not seeming to benefit, or cessation of development activities altogether.

### Statistical methods

Sample sizes for TB phase IIB trials have varied from 60 to 100 per arm, with this number considered to be adequate to demonstrate important differences in intermediate endpoints such as time to culture conversion or slope of decline in bacillary load [23, 38, 40]. We therefore selected this range for a STEP trial to explore the operating characteristics for the important secondary outcome of an unfavourable outcome. This allows us to show what could be achieved without an increase in sample size as compared to a phase IIB trial. Inference from this secondary outcome is considered first from a more familiar classical frequentist perspective and second from a Bayesian framework, which lends itself more to the sort of decision-making necessary for clinical development of regimens.

In the frequentist framework, hypothesis testing for the secondary outcome of an unfavourable outcome was set up as a 90 % two-sided confidence interval to exclude a specified proportion at the upper bound. The power for a given sample size, the proportion to be excluded at the upper bound, and the assumed true proportion were estimated from standard sample size formulae for a one-sample proportion [41]. A 90 % confidence interval (corresponding to a 5 % one-sided level of significance) was chosen given that this is a phase II trial with the objective of collecting evidence to guide decisions for future confirmatory trials, although other TB phase II trials more often use 95 % confidence intervals [24].

In the Bayesian framework, the posterior distribution of the proportion of unfavourable outcomes was calculated using a binomial distribution for the observed data and using both flat and sceptical beta conjugate priors. A flat prior assigns equal (low) probability for each unfavourable outcome proportion from 0 % to 100 %. The sceptical prior was chosen as having a fairly wide distribution with a higher density around a 20 % proportion of unfavourable outcomes, which would be an unsuitable regimen for evaluation in a phase III trial, and a lower density for very high proportions of unfavourable outcomes (extremely unlikely for a regimen selected for phase II evaluation). A prediction of the number of unfavourable outcomes for a future phase III trial was calculated from the predictive posterior distribution, which follows a beta-binomial distribution [42]. The predictive probability mimics the clinical decision-making process and provides the probability that a regimen will have an acceptably low proportion of unfavourable outcomes in a future phase III trial. The hypothetical phase III trial is said to have 500 patients per arm, which is not dissimilar to other previous and ongoing TB phase III trials.

In order to further explore the operating characteristics of the STEP trial design, data were repeatedly sampled without replacement from the recently completed RIFAQUIN phase III trial, with the unfavourable outcome as defined in the trial, censored at 12 months from randomisation. RIFAQUIN evaluated two novel regimens for the treatment of TB in seven sites across southern Africa as compared to the standard 6-month control [8]. RIFAQUIN included one arm that was shown to be non-inferior to the control regimen (the Successful Regimen) and one that did not meet criteria for non-inferiority (the Inferior Regimen). The Successful Regimen in RIFAQUIN was actually a 6-month regimen, but for the purposes of illustration, we can assume that both experimental arms were 4-month regimens. This trial is thus useful for exploring what additional information a STEP trial might have added had it been conducted before the phase III trial.

### Ethical permissions

The trial data from the RIFAQUIN trial used for the simulation study were fully anonymised for the purposes of these analyses and therefore no further ethical approval was required. The RIFAQUIN trial received ethical approval and all patients gave informed consent, as detailed in the primary publication [8].

## Results

### Power calculations in a frequentist framework

**Fig. 2:**
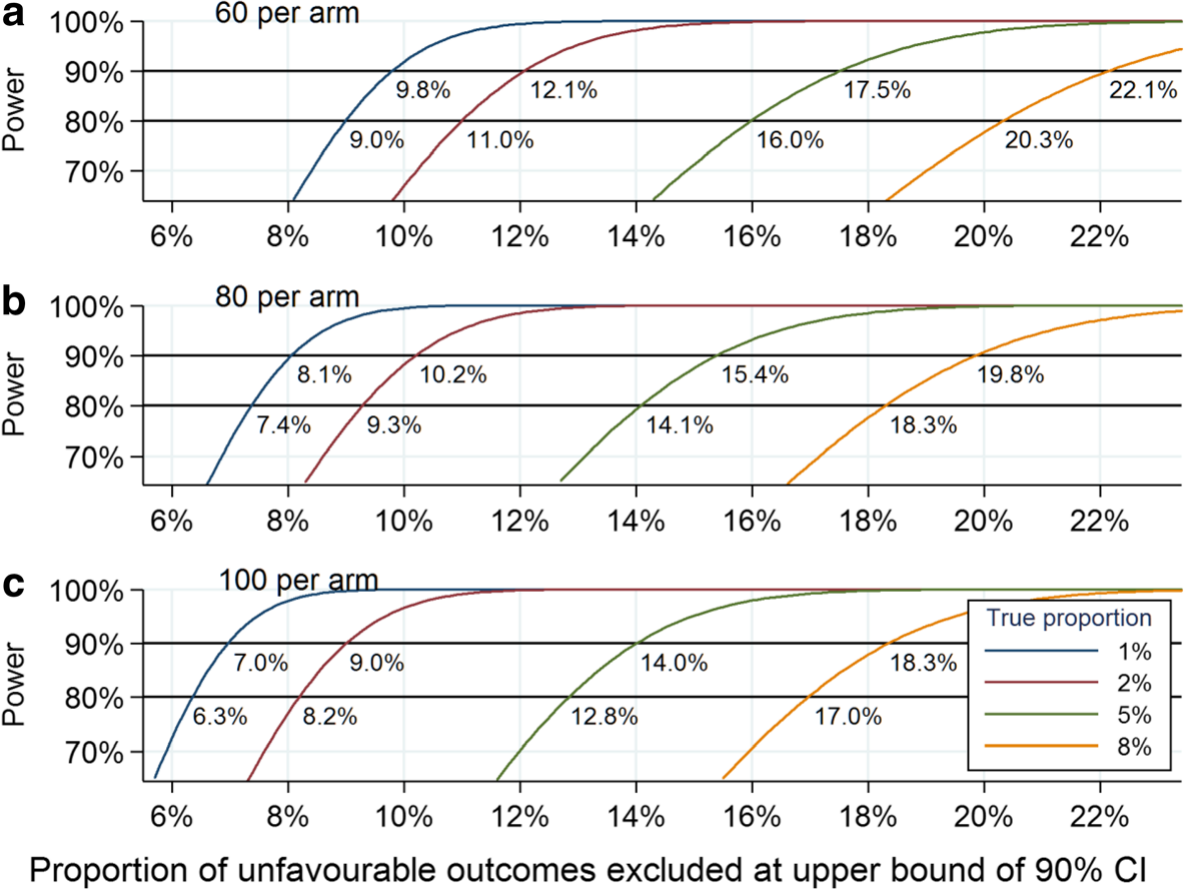
Power curves for excluding a proportion of unfavourable outcomes under different assumptions of the true proportion, assuming (**a**) 60, (**b**) 80, and (**c**) 100 patients per arm for a STEP trial. Numbers on graphs show the proportion of unfavourable outcomes that could be excluded at the upper bound of the 90 % two-sided confidence interval (95 % one-sided confidence interval, *CI*) with 80 % and 90 % power

Figure 2 shows the power curves for the secondary outcome of an unfavourable outcome under various scenarios within the frequentist framework. With a STEP trial of 80 assessable patients per arm (Fig. 2b), there would be 80 % power to exclude a proportion of unfavourable outcomes of 18.3 % at the upper bound of the 90 % confidence interval, or 90 % power to exclude a proportion of 19.8 % assuming a true proportion of unfavourable outcomes of 8 %, similar to that observed with the 6-month control regimen in recent phase III trials [1]. The same trial would give 80 % power to exclude a proportion of unfavourable outcomes of 9.3 % and 90 % power to exclude a proportion of 10.2 % for a superior regimen with a true unfavourable outcome of 2 %.

### Bayesian predictive probability

**Fig. 3:**
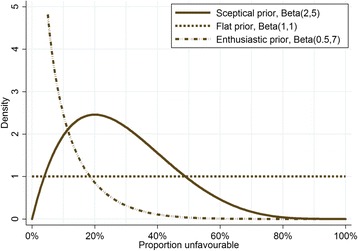
Distribution functions under various prior assumptions. The flat prior assigns equal (low) probability for each unfavourable outcome proportion from 0 % to 100 %. The sceptical prior assigns a higher probability to a 20 % proportion of unfavourable outcomes (a regimen that would not be suitable for evaluation in a phase III trial), and a lower probability to very high or very low proportions of unfavourable outcomes. The enthusiastic prior assigns a high probability to proportions of unfavourable outcomes less than 10 % and lower probabilities to proportions greater than 10 %. The sceptical prior has a median at 26.4 % and the enthusiastic prior has a median at 3.3 %

**Fig. 4:**
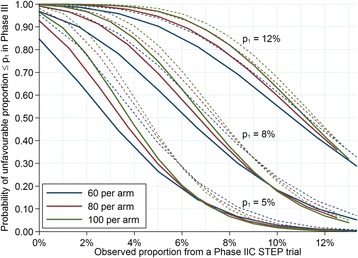
Bayesian predictive probability of the unfavourable outcome being less than a specific threshold, p_1_, in a future phase III trial of 500 patients per arm, following various observed proportions of unfavourable outcomes and patient numbers in a STEP trial of equal treatment duration. *Solid lines* correspond to a sceptical prior and *dashed lines* to the flat prior

An alternative perspective would be to consider the Bayesian predictive probability of the unfavourable outcome being lower than a pre-specified threshold (p_1_) in a future phase III trial with 500 patients per arm, given the observed unfavourable outcome from the STEP trial and incorporating prior information. This approach allows for explicit incorporation of the prior confidence in a regimen before the data are collected in the STEP trial. Figure 3 shows three different prior distributions reflecting a range of prior confidence from sceptical to enthusiastic. Figure 4 shows the Bayesian predictive probability that the proportion of unfavourable outcomes is less than a pre-specified threshold (p_1_) in a potential phase III trial given a sceptical or flat prior. With the sceptical prior and 80 patients per arm, two (2.5 %) unfavourable outcomes in the STEP trial would mean there is a probability of 0.99 that the proportion of unfavourable outcomes in the potential phase III would be less than p_1_ = 12 % and a probability of 0.91 that the proportion of unfavourable outcomes would be less than p_1_ = 8 %. With six (7.5 %) unfavourable outcomes in the STEP trial, there is a probability of 0.82 that the proportion of unfavourable outcomes in a potential phase III would be less than p_1_ = 12 % and a probability of 0.41 that it would be less than p_1_ = 8 %.

### Results from simulations employing the RIFAQUIN trial data

**Fig. 5:**
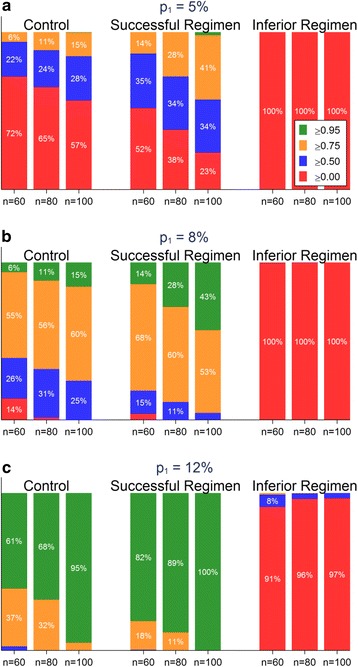
Results of a simulation study using RIFAQUIN data with a sceptical prior. Charts show the distribution of the predictive probability of the unfavourable proportion in the phase III trial being lower than the threshold p_1_ from 10,000 random draws from the RIFAQUIN trial data for various sizes of STEP trials (60, 80, 100 per arm) and thresholds p_1_ = 5 % (**a**), 8 % (**b**), and 10 % (**c**)

**Fig. 6:**
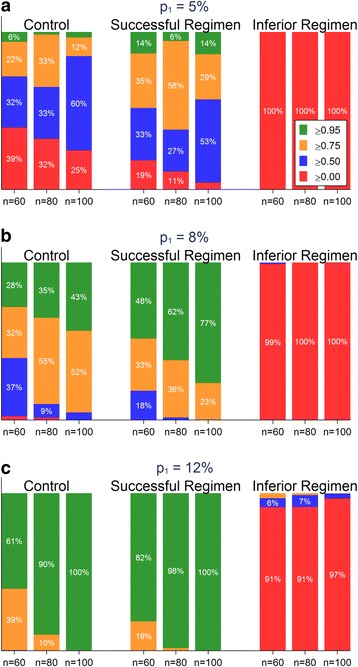
Results of a simulation study using RIFAQUIN data with a flat prior. Charts show the distribution of the predictive probability of the unfavourable proportion in the phase III trial being lower than the threshold p_1_ from 10,000 random draws from the RIFAQUIN trial data for various sizes of STEP trials (60, 80, 100 per arm) and thresholds p_1_ = 5 % (**a**), 8 % (**b**), and 10 % (**c**)

**Fig. 7:**
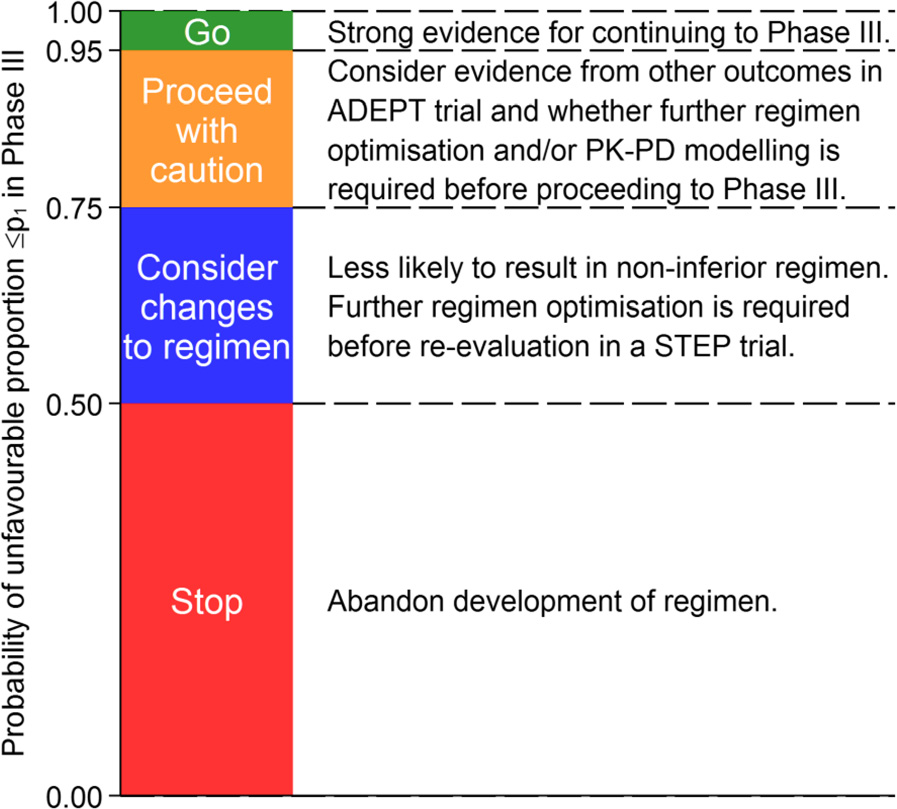
Traffic light system for interpreting the Bayesian predictive probability

Sampling data from the RIFAQUIN trial allows us to consider what the additional benefit of a STEP trial would have been had it been conducted before the phase III trial, showing how such a trial design might be used in practice. Focusing specifically on the Bayesian formulation, Fig. 5 shows the results of 10,000 random draws from the RIFAQUIN trial data using the primary outcome as defined in the trial censored at 12 months [8] using a sceptical prior. Similar results with the flat prior are shown in Fig. 6. The predictive probability is grouped into four categories to show possible decision outcomes: 0.00 to <0.50, 0.50 to <0.75, 0.75 to <0.95, and 0.95 to 1.00 (Fig. 7). A predictive probability of <0.50 might be used to indicate that the development of a regimen be abandoned (*stop*, red bars) and a predictive probability of >0.95 might be used to indicate strong evidence for continuing the regimen to a phase III trial (*go*, green bars). Predictive probabilities of 0.75 to <0.95 (*proceed with caution*, amber bars) might be used to indicate further optimisation is required before proceeding to evaluation in a phase III trial, with the decision depending on other outcomes from the STEP trial. Predictive probabilities of 0.50 to <0.75 (blue bars) might be used to indicate changes to the regimen should be considered before possible further re-evaluation in a STEP trial.

Considering a STEP trial of 80 patients per arm, 100 % of the simulated trials for the Inferior Regimen had a predictive probability of less than 0.50 that the unfavourable proportion in a phase III trial would be less than p_1_ = 8 % (Fig. 5b) indicating this regimen should be abandoned (red bars). In contrast, 28 % of the simulated trials for the Successful Regimen had a predictive probability of greater than 0.95 that the unfavourable proportion in a phase III trial would be less than p_1_ = 8 % (green bars), and 60 % for the Successful Regimen had a predictive probability between 0.75 and 0.95 (amber bars). This shows a STEP trial would have correctly shown that the Inferior Regimen should not proceed to phase III (this regimen was shown to have significantly more unfavourable outcomes than the control arm in the RIFAQUIN phase III trial). It would also have had a high chance (88 %) of either showing that the Successful Regimen could proceed to phase III or that it might require further optimisation (the Successful Regimen was shown to be non-inferior to control in the RIFAQUIN phase III trial). Using a larger STEP trial with 100 patients per arm results in a greater chance of a predictive probability of more than 0.95 (43 % of trials), as would using a flat prior instead of a sceptical prior (Fig. 6).

## Discussion

We have described how a trial with the novel Phase IIC STEP design and a similar sample size to that commonly used in phase IIB trials for TB regimens could be used to provide important additional information to inform the decision on whether to proceed to evaluation in phase III. Furthermore, a simulation study using data from the RIFAQUIN trial demonstrated that conducting a STEP trial first with 80 patients per arm would have led to the correct decision in 100 % of the simulated trials to not proceed with evaluation in a phase III trial with a regimen that was subsequently shown to be inferior, saving cost and reducing risk to participants. Use of such a design could fundamentally improve our ability to choose those regimens most likely to succeed in phase III evaluation and help us avoid costly phase III failures.

We favour a Bayesian approach to the interpretation of a STEP trial, but have also presented the frequentist framework to show how this could be applied. The Bayesian probability is easy to interpret and fits more naturally into a decision framework for deciding whether to move a regimen into phase III evaluation or whether a regimen requires further optimisation. A traffic light system for ‘stop’, ‘go,’ or ‘proceed with caution’ might be used to improve understanding of the results. A probability of at least 0.95 for the unfavourable proportion (p_1_) being less than 8 % might be considered a reasonable threshold for progressing to a phase III trial, which would be analogous to the 95 % confidence intervals and 5 % type I error rate commonly adopted in frequentist hypothesis testing.

In principle, STEP trial results could be used to support subsequent trials of a different duration than that which was studied in the STEP trial itself. A very high predictive probability might indicate a shorter regimen could be considered for phase III (a 3-month regimen if a 4-month regimen was studied in the STEP trial, for example), and a more modest predictive probability might indicate a longer regimen should be considered for phase III. While this paper focuses on the development of regimens for drug-sensitive TB, a STEP trial would also be suitable for regimens for MDR-TB where 6-month or 9-month regimens are tested.

The Bayesian framework allows for the incorporation of prior information. We have used and would recommend a slightly sceptical prior rather than a flat, so-called non-informative prior because this more accurately reflects the likelihood of a new regimen failing in clinical development. Our chosen sceptical prior assumes that an unfavourable proportion of around 20 % is most likely, but has a wide distribution and therefore has only limited impact on the interpretation of the results (shown by the closeness of the lines assuming the sceptical and flat priors in Fig. 4). However, more sophisticated priors could be used to formally incorporate promising preclinical as well as any other earlier clinical trial data on efficacy into the decision-making process. The beta prior lends itself to this sort of approach, where the distributional parameters *a* and *b* can be interpreted as the strength of real data with *a* unfavourable outcomes and *b* favourable outcomes. For example, a relapse murine study of 15 mice with only one relapse and 14 cures could be summarised as a prior with a Beta(0.5, 7) distribution (the enthusiastic prior in Fig. 3), if considering data from a murine study as having half the strength of a human study (*a* = 1/2 = 0.5, and *b* = 14/2 = 7). In such a situation, it would be appropriate to conduct the analysis using both the sceptical and enthusiastic priors and compare the results.

The results regarding the secondary outcome (an unfavourable outcome) must be considered alongside the results of the primary outcome (killing of bacilli) when deciding how to proceed with the regimen under evaluation. If the predictive probability was greater than 0.75, a large increase in killing of bacilli with a new regimen would strengthen the decision to move to phase III whereas only a modest increase would provide evidence that further optimisation was needed. The results regarding the intermediate outcome could also be formally incorporated into the decision process by updating the prior with this information in conjunction with a prediction model that links the intermediate outcome with the long-term clinical outcome (one published model uses 2-month culture results on solid media [43]). The primary intermediate outcome data would likely be available before the long-term clinical unfavourable outcome data, so this updating of the prior could be done after that primary outcome analysis and before the long-term clinical data were available.

There are a number of factors that will determine whether a STEP trial could replace a traditional phase IIB trial in TB regimen development. It would be essential to have reasonable confidence that the drug combination could be used safely for 3 or 4 months, based on the adverse event profile and knowledge about the relationship between the duration of treatment and risk of toxicity. It is also necessary to have sufficient confidence in the efficacy of a regimen and the capacity of the trial sites to follow up patients to be secure in stopping treatment after 3 or 4 months; otherwise, trial participants are put at unnecessary risk of relapse, with its resultant morbidity. Where confidence is insufficient to proceed directly to a STEP trial based on available knowledge after a smaller 14-day study, a STEP trial might still be considered after the phase IIB trial owing to the benefits in preventing costly phase III failure. Where the evidence preceding a STEP trial may not be as strong as that preceding a large phase III trial, some arms might be at unusually high risk of failure and early relapse. In such cases, careful patient monitoring (including regular cultures during treatment) and frequent and efficient oversight by an independent data monitoring committee are important features.

We believe that the richness of data from a STEP trial supports a broader philosophy in the middle development of ‘iterative optimisation’ where the objective of efficiently selecting from among numerous optimised combination regimens is met. As combinations are shown to be effective and safe, newer drugs can be added, doses adjusted (making fullest use of PK-PD modelling), and drugs replaced in sequential STEP evaluation (or phase IIB where necessary) until a regimen is identified that is sufficiently promising to proceed to evaluation in phase III. Indeed, competing combinations can be evaluated side-by-side within the same multi-arm trial. A MAMS design [7] would be appropriate to allow recruitment to unpromising arms to be stopped early, taking into account long-term clinical outcomes and short-term microbiological outcomes, thereby enhancing efficiency. The flexibility of the MAMS design also allows for adding new arms so that sequential evaluations could be managed within the same platform trial, an approach that has been successful in prostate cancer trials [44].

There are a number of limitations with a STEP trial. Additional follow-up visits are required after the end of treatment and these incur costs and increase the duration of the trial by 6 months, thereby delaying the start of the phase III trial. However, a visit at the end of treatment, at 12 months post-randomisation, and a third intermediate visit, with sputum taken for culture at each visit, would likely be adequate to identify treatment failure and relapse and determine 12-month cure. The simulation study using the RIFAQUIN trial data also indicated that a slightly larger sample size of 100 per arm might be preferable for a STEP trial to give more information and greater confidence in decision-making for phase III.

More work is needed to consider further extensions of the methodology and how this might operate in practice. A Bayesian adaptive trial design could also be used where the prior is continually updated as data accrue on both the primary intermediate outcome and the long-term unfavourable outcome during the STEP trial, with rules for stopping arms early. This would allow for real-time assessment of risks and benefits to participants [42]. The Bayesian framework has been set up around the predictive probability of the unfavourable proportion being lower than a pre-specified threshold (p_1_) in a future phase III trial, but a more practical alternative would be to incorporate results on the control arm from the STEP trial and calculate the unconditional probability of actually demonstrating non-inferiority in a future phase III. This is known as ‘assurance’, which is a Bayesian extension of the concept of the power of a clinical trial that more formally incorporates the uncertainty resulting from a phase II trial [45].

The iterative approach to regimen development, in contrast to the linear approach, may be of most value when there are multiple promising drugs and combinations to be evaluated in middle development so that the most promising regimen or regimens can be taken forward to phase III. In practice, this may be limited by the extent to which individual drugs can be evaluated in combination prior to regulatory approval and the limitations imposed by regulators and the companies which own the intellectual property. These limitations must be overcome in order to improve the treatment of TB and are being addressed in other arenas, an example being the 3P Project [46].

We have proposed a traffic light system for the predictive probability. Our intention was to set a high bar for moving regimens forward to phase III trials, given that the STEP trial design was motivated by the desire to reduce the risk of costly phase III failures. For this reason, the relatively small sample size with the STEP trial design means that there is a higher chance of a false negative result, where a truly effective regimen is not immediately advanced to phase III. Simulations from the RIFAQUIN trial showed that only 28 % of STEP trials with 80 patients per arm would have resulted in a predictive probability of greater than 0.95. Further work is needed to evaluate whether the 0.95 threshold could be relaxed; one approach would be to consider how this impacts frequentist concepts like power and type I error rate [47]. However, the Bayesian approach is particularly useful here because it quantifies the likelihood of phase III success, rather than imposing a dichotomous go/no-go decision. As we have shown, a predictive probability of less than 0.95 need not necessarily lead to a regimen being abandoned, but would suggest that the regimen may require further optimisation before proceeding to phase III. In this setting, critical evaluation of all the trial outcomes (not just the primary endpoint) and of variability in measurement of the primary endpoint would be essential, as would sophisticated PK-PD analyses (to see if higher exposures improve outcomes). A STEP trial may have delayed the start of the RIFAQUIN trial, but we submit that this would have been a worthwhile risk given that it would have shown that the Inferior Regimen should not have been advanced.

## Conclusions

We have proposed the Phase IIC STEP design that (1) yields rich, highly relevant information about treatment outcomes that is additional and complementary to the intermediate microbiological data typically gathered in phase II trials and (2) facilitates iterative optimisation of combination regimens in middle development. We have shown that collecting definitive clinical outcome data in a relatively small number of participants over only 12 months does provide invaluable information about the likelihood of success in a future phase III trial. For these reasons, we strongly believe that the STEP design described herein is an important tool for use in middle development for new TB regimens that would allow for more informed decision-making and accelerate regimen development.

### Availability of data and materials

The data from the RIFAQUIN trial used for these analyses is available for eligible researchers as part of a repository of TB trial data. See http://c-path.org/programs/tb-pacts/ for further details.
